# Supplementary material for: Unveiling the Bioactive Potential of the Invasive Jellyfish Phyllorhiza punctata Through Integrative Transcriptomic and Proteomic Analyses
Source: Biomolecules. 2025 Aug 4;15(8):1121. doi: 10.3390/biom15081121 (PMC12383608; doi:10.3390/biom15081121)
Supplement: Supplementary file 1 [file biomolecules-15-01121-s001.zip › Suplementary Material/FileS3/PEPFOLD4/XP_065065861_3565/clusters.html]

 PEP-FOLD clusters


Download archive of all models  here   

|  |  |  |
| --- | --- | --- |
| # | file | sOPEP |
| # cluster 1 - Size: 9 |  |  |
|  | XP\_065065861\_3565-model1 | -14.1718 |
|  | XP\_065065861\_3565-model1.1 | -14.094 |
|  | XP\_065065861\_3565-model1.2 | -14.094 |
|  | XP\_065065861\_3565-model1.3 | -14.094 |
|  | XP\_065065861\_3565-model1.4 | -14.094 |
|  | XP\_065065861\_3565-model1.5 | -14.094 |
|  | XP\_065065861\_3565-model1.6 | -14.0302 |
|  | XP\_065065861\_3565-model1.7 | -13.9101 |
|  | XP\_065065861\_3565-model1.8 | -13.8655 |
| # cluster 2 - Size: 11 |  |  |
|  | XP\_065065861\_3565-model2 | -14.1358 |
|  | XP\_065065861\_3565-model2.1 | -14.1358 |
|  | XP\_065065861\_3565-model2.2 | -14.1358 |
|  | XP\_065065861\_3565-model2.3 | -13.9686 |
|  | XP\_065065861\_3565-model2.4 | -13.9686 |
|  | XP\_065065861\_3565-model2.5 | -13.9686 |
|  | XP\_065065861\_3565-model2.6 | -13.9686 |
|  | XP\_065065861\_3565-model2.7 | -13.9686 |
|  | XP\_065065861\_3565-model2.8 | -13.9686 |
|  | XP\_065065861\_3565-model2.9 | -13.9686 |
|  | XP\_065065861\_3565-model2.10 | -13.9686 |
| # cluster 3 - Size: 6 |  |  |
|  | XP\_065065861\_3565-model3 | -14.1024 |
|  | XP\_065065861\_3565-model3.1 | -14.1024 |
|  | XP\_065065861\_3565-model3.2 | -14.1024 |
|  | XP\_065065861\_3565-model3.3 | -13.9579 |
|  | XP\_065065861\_3565-model3.4 | -13.9579 |
|  | XP\_065065861\_3565-model3.5 | -13.9096 |
| # cluster 4 - Size: 5 |  |  |
|  | XP\_065065861\_3565-model4 | -14.0973 |
|  | XP\_065065861\_3565-model4.1 | -13.9292 |
|  | XP\_065065861\_3565-model4.2 | -13.9273 |
|  | XP\_065065861\_3565-model4.3 | -13.9273 |
|  | XP\_065065861\_3565-model4.4 | -13.8513 |
| # cluster 5 - Size: 5 |  |  |
|  | XP\_065065861\_3565-model5 | -14.0515 |
|  | XP\_065065861\_3565-model5.1 | -14.0515 |
|  | XP\_065065861\_3565-model5.2 | -14.0515 |
|  | XP\_065065861\_3565-model5.3 | -14.0515 |
|  | XP\_065065861\_3565-model5.4 | -13.9439 |
| # cluster 6 - Size: 20 |  |  |
|  | XP\_065065861\_3565-model6 | -14.0419 |
|  | XP\_065065861\_3565-model6.1 | -14.0224 |
|  | XP\_065065861\_3565-model6.2 | -14.0224 |
|  | XP\_065065861\_3565-model6.3 | -14.0224 |
|  | XP\_065065861\_3565-model6.4 | -14.0224 |
|  | XP\_065065861\_3565-model6.5 | -14.0224 |
|  | XP\_065065861\_3565-model6.6 | -14.0224 |
|  | XP\_065065861\_3565-model6.7 | -14.0224 |
|  | XP\_065065861\_3565-model6.8 | -14.0224 |
|  | XP\_065065861\_3565-model6.9 | -14.0224 |
|  | XP\_065065861\_3565-model6.10 | -14.0224 |
|  | XP\_065065861\_3565-model6.11 | -14.0224 |
|  | XP\_065065861\_3565-model6.12 | -14.0224 |
|  | XP\_065065861\_3565-model6.13 | -14.0224 |
|  | XP\_065065861\_3565-model6.14 | -14.0224 |
|  | XP\_065065861\_3565-model6.15 | -13.9315 |
|  | XP\_065065861\_3565-model6.16 | -13.9315 |
|  | XP\_065065861\_3565-model6.17 | -13.9315 |
|  | XP\_065065861\_3565-model6.18 | -13.9315 |
|  | XP\_065065861\_3565-model6.19 | -13.9315 |
| # cluster 7 - Size: 27 |  |  |
|  | XP\_065065861\_3565-model7 | -14.037 |
|  | XP\_065065861\_3565-model7.1 | -14.037 |
|  | XP\_065065861\_3565-model7.2 | -14.037 |
|  | XP\_065065861\_3565-model7.3 | -14.037 |
|  | XP\_065065861\_3565-model7.4 | -14.037 |
|  | XP\_065065861\_3565-model7.5 | -14.037 |
|  | XP\_065065861\_3565-model7.6 | -14.037 |
|  | XP\_065065861\_3565-model7.7 | -14.037 |
|  | XP\_065065861\_3565-model7.8 | -14.037 |
|  | XP\_065065861\_3565-model7.9 | -14.037 |
|  | XP\_065065861\_3565-model7.10 | -14.037 |
|  | XP\_065065861\_3565-model7.11 | -14.037 |
|  | XP\_065065861\_3565-model7.12 | -14.037 |
|  | XP\_065065861\_3565-model7.13 | -14.037 |
|  | XP\_065065861\_3565-model7.14 | -14.037 |
|  | XP\_065065861\_3565-model7.15 | -14.037 |
|  | XP\_065065861\_3565-model7.16 | -13.9922 |
|  | XP\_065065861\_3565-model7.17 | -13.9922 |
|  | XP\_065065861\_3565-model7.18 | -13.9922 |
|  | XP\_065065861\_3565-model7.19 | -13.9922 |
|  | XP\_065065861\_3565-model7.20 | -13.9922 |
|  | XP\_065065861\_3565-model7.21 | -13.9922 |
|  | XP\_065065861\_3565-model7.22 | -13.9922 |
|  | XP\_065065861\_3565-model7.23 | -13.9922 |
|  | XP\_065065861\_3565-model7.24 | -13.9922 |
|  | XP\_065065861\_3565-model7.25 | -13.9922 |
|  | XP\_065065861\_3565-model7.26 | -13.9255 |
| # cluster 8 - Size: 3 |  |  |
|  | XP\_065065861\_3565-model8 | -14.008 |
|  | XP\_065065861\_3565-model8.1 | -14.008 |
|  | XP\_065065861\_3565-model8.2 | -13.8038 |
| # cluster 9 - Size: 3 |  |  |
|  | XP\_065065861\_3565-model9 | -14.0047 |
|  | XP\_065065861\_3565-model9.1 | -14.0047 |
|  | XP\_065065861\_3565-model9.2 | -13.932 |
| # cluster 10 - Size: 4 |  |  |
|  | XP\_065065861\_3565-model10 | -13.9895 |
|  | XP\_065065861\_3565-model10.1 | -13.9895 |
|  | XP\_065065861\_3565-model10.2 | -13.9713 |
|  | XP\_065065861\_3565-model10.3 | -13.8852 |
| # cluster 11 - Size: 3 |  |  |
|  | XP\_065065861\_3565-model11 | -13.9506 |
|  | XP\_065065861\_3565-model11.1 | -13.8524 |
|  | XP\_065065861\_3565-model11.2 | -13.8125 |
| # cluster 12 - Size: 2 |  |  |
|  | XP\_065065861\_3565-model12 | -13.948 |
|  | XP\_065065861\_3565-model12.1 | -13.948 |
| # cluster 13 - Size: 1 |  |  |
|  | XP\_065065861\_3565-model13 | -13.8725 |
| # cluster 14 - Size: 1 |  |  |
|  | XP\_065065861\_3565-model14 | -13.7835 |
|  |  |  |
